# Flexural-strength screening and acid-challenge response of bovine hydroxyapatite formulations with zirconia-based additions under formulation-specific sintering

**DOI:** 10.3389/fdmed.2026.1940170

**Published:** 2026-10-21

**Authors:** Lucas José de Azevedo-Silva, Brunna Mota Ferrairo, Lucas Yoshizawa de Marins, Marina Ciccone Giacomini, Paulo Noronha Lisboa-Filho, Ana Flávia Sanches Borges, Carlos Alberto Fortulan

**Affiliations:** 1 Department of Prosthodontics and Periodontics, Faculty of Dentistry of Bauru, University of São Paulo, Bauru, Brazil; 2 Department of Operative Dentistry, Endodontics and Dental Materials, Faculty of Dentistry of Bauru, University of São Paulo, Bauru, Brazil; 3 Department of Physics, School of Sciences, São Paulo State University, Bauru, Brazil; 4 Department of Mechanical Engineering, São Carlos School of Engineering, University of São Paulo, São Carlos, Brazil

**Keywords:** 3Y-TZP, calcium phosphates, ceramic composites, chemical solubility, dental bioceramics, hydroxyapatite, Vickers hardness, zirconia

## Abstract

Hydroxyapatite-based (HA) bioceramics are attractive for dental-biomaterial development, but their mechanical and chemical limitations remain challenging. This study screened bovine-HA formulation-processing routes with zirconia-based additions by three-point flexural strength and then assessed chemical solubility, Vickers hardness, and qualitative surface morphology of the mechanically selected route after an acidic challenge. In Phase 1, pure HA and HA containing 1, 5, or 10 wt% 3Y-TZP or an exploratory nominal ZrO_2_ whisker-synthesis product were evaluated under formulation-specific sintering schedules. Overall flexural-strength differences were significant (*p* < 0.001). HA + 1 wt% 3Y-TZP showed the highest observed median [117.0 (94.8, 122.0) MPa], although its pairwise comparison with pure HA [84.0 (77.0, 88.0) MPa] did not reach significance (DSCF *p* = 0.095). In Phase 2, aggregate chemical solubility was 2,112.5 μg/cm^2^ for pure HA and 2,057.0 μg/cm^2^ for HA + 1 wt% 3Y-TZP; both exceeded the <2,000 μg/cm^2^ limit applicable to covered substructure ceramics in ISO 6872:2024 Classes 2–4, while monolithic ceramics are subject to the more stringent <100 μg/cm^2^ limit. HA + 1 wt% 3Y-TZP showed higher Vickers hardness than pure HA before (435.9 ± 26.4 vs. 217.0 ± 5.6 HV0.3) and after testing (426.9 ± 38.9 vs. 196.7 ± 10.5 HV0.3; Bonferroni-adjusted *p* < 0.001 for both). Pure HA showed a within-route decrease of −20.3 HV0.3 (95% CI: −33.1 to −7.5; adjusted *p* = 0.048), whereas the selected route showed a change of −9.0 HV0.3 (95% CI: −44.3 to 26.4; adjusted *p* = 1.000). Direct comparison of the changes was not significant (difference, −11.3 HV0.3; 95% CI: −46.7 to 24.1; adjusted *p* = 1.000), so differential hardness resistance to the acidic challenge was not demonstrated. Because composition and thermal history were not independently controlled, the findings should be interpreted as route-level screening rather than as an isolated zirconia effect or clinical validation.

## 1 Introduction

Calcium phosphate bioceramics are central to biomedical and dental materials research because their composition and biological behavior are closely related to the mineral phase of hard tissues. Among them, hydroxyapatite (HA) is particularly attractive due to its compositional similarity to bone and dental mineral tissues. However, despite its biological relevance, dense HA ceramics still present important mechanical limitations, including brittleness, low fracture resistance, and high sensitivity to porosity, grain growth, densification, and phase stability (1). These limitations restrict the use of dense HA in mechanically demanding conditions and support the development of reinforced HA-based systems with improved mechanical and physicochemical performance.

Natural-source HA has gained attention as an alternative route for producing calcium phosphate bioceramics. Animal bones are calcium-phosphate-rich by-products that can be processed as a source of hydroxyapatite, providing a material-recovery pathway for this mineral-rich biomass (2, 3). Previous investigations by the present group established thermochemical and mechanochemical routes for obtaining bovine-derived HA nanopowders and dense HA-based bioceramics (4–6). Using the same starting powder lot as the present investigation, Azevedo-Silva et al. (7) evaluated bovine HA containing 0, 1, 5, or 10 wt% 3Y-TZP under three sintering curves. That study identified conventional sintering at 1,300 °C for pure HA and a two-step schedule reaching 1,420 °C for the 3Y-TZP-containing formulations as the most favorable composition-specific processing routes. XRD showed characteristic HA reflections in pure HA and HA + 1 wt% 3Y-TZP, whereas HA decomposition to β-TCP and calcium zirconate formation were detected at 5 and 10 wt% 3Y-TZP. Pereira et al. (8) subsequently applied the ISO 6872 chemical-solubility and before/after hardness workflow to TiO_2_-modified bovine HA and identified chemical durability as a persistent limitation. The present study used newly manufactured specimens; no specimens or outcome data from Azevedo-Silva et al. (7) were reused. Its incremental contribution is an independent ISO-bar three-point flexural-strength screening of zirconia-based routes, including an exploratory nominal W-ZrO_2_ route, followed by chemical-solubility and repeated-hardness assessment of the mechanically selected zirconia-containing formulation-processing route.

Zirconia is a widely used structural bioceramic in dentistry because of its high mechanical strength, chemical stability, biocompatibility, and transformation-toughening potential (9–11). Yttria-stabilized tetragonal zirconia polycrystal (Y-TZP) is used in several dental applications because of its high flexural strength and fracture resistance, although phase stability and low-temperature degradation remain important considerations for zirconia-based materials (10, 12). In HA-based composites, zirconia may act as a reinforcing phase; however, this effect is not necessarily linear or concentration-dependent. HA–zirconia systems are sensitive to high-temperature processing, and previous studies have reported that zirconia addition may influence HA decomposition, secondary phase formation, porosity, and densification depending on the composition and sintering conditions (13, 14).

In addition to particle reinforcement, whisker-based strategies have been investigated to modify fracture behavior in brittle ceramics through crack deflection, crack bridging, and pull-out (15, 16). These mechanisms require verified whisker morphology, adequate aspect ratio and dispersion, and appropriate interfacial bonding. In the present study, the molten-salt synthesis product was prepared according to a published zirconia-whisker route, but its phase and aspect ratio were not independently characterized; it is therefore referred to as the nominal ZrO_2_ whisker-synthesis product. Its comparison with 3Y-TZP particles is interpreted as exploratory screening of two processing routes, not as a definitive test of whisker toughening. For dental material development, mechanical screening must also be coupled with standardized chemical-durability and surface-response assessments.

The rationale was to extend the authors’ prior composition-sintering optimization using newly manufactured specimens and a distinct sequential screening strategy: first, to rank bovine HA formulation-processing routes containing 3Y-TZP or an exploratory nominal W-ZrO_2_ synthesis product by three-point flexural strength in ISO bar geometry; and second, to characterize chemical solubility descriptively and compare Vickers hardness of the mechanically selected route and the pure-HA route before and after a standardized acidic challenge. The study did not aim to identify phase constitution, quantify microstructure, or measure fracture toughness. Accordingly, the route with the highest observed median flexural strength was selected for Phase 2 and compared with pure HA regarding aggregate chemical solubility, specimen-level Vickers hardness, and qualitative SEM surface appearance. The null hypothesis was that the tested formulation-processing groups would not differ in flexural strength and that the selected route would not differ from the pure-HA route in Vickers hardness. Chemical solubility was treated descriptively because each group contributed one jointly weighed 11-disk gravimetric set.

## 2 Materials and methods

### 2.1 Experimental design

This study was designed as a two-phase *in vitro* investigation of formulation-processing routes. All specimens reported here were newly pressed and sintered from the same starting powder lot used by Azevedo-Silva et al. (7), following the same powder-processing and formulation-specific sintering methodology; none of the specimens or outcome measurements from the prior publication were reused. In Phase 1, seven groups were screened by three-point flexural strength: pure HA; HA + 1, 5, or 10 wt% 3Y-TZP; and HA + 1, 5, or 10 wt% nominal ZrO_2_ whisker-synthesis product. In Phase 2, the route with the highest observed median flexural strength was compared with the pure-HA route regarding chemical solubility and Vickers hardness before and after chemical solubility testing. SEM was used only for qualitative description of representative surface fields. Because pure HA and zirconia-containing formulations were processed with different schedules, the study estimates differences among complete formulation-processing routes; it does not independently estimate a composition-only effect.

### 2.2 Preparation of HA nano-powder

Bovine HA powder was obtained from bovine bone tissue according to previously described thermo and mechanochemical protocols (5, 6). Briefly, bovine bone tissue was subjected to thermochemical treatment using hydrogen peroxide (H_2_O_2_, 100 volumes), followed by calcination at 900 °C for 24 h to remove organic constituents. The resulting HA was ground to reduce particle size, increase interparticle reactivity, and favor subsequent ceramic consolidation.

For nanoparticulation, HA powder was placed in a polyethylene jar containing 3Y-TZP zirconia milling media. The jar was loaded with a suspension composed of 30 vol% HA, 69.95 vol% isopropyl alcohol (C₃H₈O; Synth, Brazil), and 0.05 wt% para-aminobenzoic acid (PABA; C₇H₇NO_2_; Sigma-Aldrich, USA). The suspension was first processed in a ball mill for 48 h and subsequently in a vibrating mill for 72 h, following previously established protocols for bovine HA nanopowder production (6). Potential wear-derived transfer from the 3Y-TZP milling media into the HA powder was not quantified in the present study.

### 2.3 Preparation of the ZrO_2_ whisker-synthesis product

A product intended to contain ZrO_2_ whiskers was synthesized using a molten-salt route reported for zirconia-whisker production (17). Zirconyl chloride octahydrate (ZrOCl_2_·8H_2_O; Sigma-Aldrich, USA), trisodium phosphate dodecahydrate (Na_3_PO_4_·12H_2_O; Sigma-Aldrich, USA), and sodium fluoride (NaF) were weighed at 2 g, 2 g, and 0.2 g, respectively, manually ground until homogeneous, transferred to a crucible, and calcined at 900 °C for 5 h. The material was rapidly cooled, washed three times with deionized water to remove residual salts, and dried at 70 °C for 6 h. A retained aliquot from the same synthesis batch used to fabricate the specimens was subsequently documented by SEM between May and October 2023, as described in Section 2.9. Powder XRD and quantitative dimensional analysis were not available for this batch; therefore, crystalline phase identity, verified whisker morphology, and aspect ratio were not established. The material is denoted nominal W-ZrO_2_ in group labels. The synthesis was performed once; the recovered product from this single batch was apportioned among the exploratory W-ZrO_2_ formulation groups, and a retained aliquot was later used for SEM documentation. The recovered dry mass was not recorded as an experimental outcome.

### 2.4 Preparation of HA powders with zirconia-based additions

To prepare the experimental compositions, commercial granulated 3Y-TZP powder (TZ-3YSB-E, lot SY307525B; Tosoh Corporation, Japan), containing 3 wt% pressing binder according to the manufacturer's technical specification, or the nominal W-ZrO_2_ synthesis product was separately added to HA nanopowder at nominal concentrations of 1, 5, or 10 wt%. The nominal percentages refer to the as-received 3Y-TZP powder mass and therefore include the binder fraction. Each zirconia-containing mixture was returned to the vibrating mill for an additional 24 h solely to homogenize the added powder within the HA suspension. Pure HA did not undergo this additional 24-h homogenization step. After mixing, the slurry was dried using hot air at approximately 80 °C, and the agglomerates were sieved through a ≤75 μm mesh (#200) before specimen fabrication.

### 2.5 Specimen preparation

Dense HA-based specimens were fabricated using the experimental powders described above. Bar-shaped specimens were prepared for three-point flexural strength testing, whereas disk-shaped specimens were prepared for chemical solubility, Vickers hardness, and SEM analyses. Before sintering, the powders were compacted to nominal dimensions of approximately 21 mm × 4 mm × 3 mm for bars and 15 mm in diameter × 1.4 mm in thickness for disks. These dimensions correspond to the compacted green specimens, not the final post-sintering dimensions. Specimens were first uniaxially pressed at 100 MPa for 1 min and then isostatically pressed at 200 MPa for 1 min, according to previously established protocols for dense bovine HA-based ceramics (4, 6, 7, 18, 19). For the disks subsequently used in chemical-solubility testing, measured post-sintering/pre-test dimensions were 13.00 ± 0.14 mm in diameter and 1.27 ± 0.04 mm in thickness for pure HA and 12.09 ± 0.02 mm in diameter and 1.37 ± 0.03 mm in thickness for HA + 1 wt% 3Y-TZP.

Pure HA specimens were sintered using a one-step sintering protocol. The temperature was increased from room temperature to 160 °C at 2.7 °C/min, then to 600 °C at 4 °C/min, to 1,100 °C at 5 °C/min, and finally to 1,300 °C at 6 °C/min, followed by a 120-min holding period. The specimens were then cooled to room temperature inside the furnace.

The 3Y-TZP-containing HA formulations were sintered using the two-step schedule supported by the authors' previous composition-specific optimization (7). That investigation compared conventional sintering at 1,300 °C with two-step schedules reaching 1,292 °C or 1,420 °C and supported conventional 1,300 °C processing for pure HA and the 1,420 °C two-step route for the 3Y-TZP-containing formulations. In the present study, the same two-step schedule was applied by extension to the exploratory nominal W-ZrO_2_ routes; these W-ZrO_2_ routes were not independently optimized. The temperature was increased from room temperature to 650 °C at 4 °C/min, then to 900 °C at 5 °C/min and to 1,420 °C at 10 °C/min. After a 6-min hold at 1,420 °C, the temperature was reduced to 950 °C at 10 °C/min, followed by a 20-h hold and gradual furnace cooling. No bridging control (pure HA under the two-step schedule or HA + 1 wt% 3Y-TZP under the one-step schedule) was included; composition and thermal history are therefore inseparable in between-route comparisons.

### 2.6 Three-point flexural strength

Three-point flexural strength was evaluated in all experimental groups as the mechanical screening outcome, according to ISO 6872:2024 for dental ceramic materials. Ten bar-shaped specimens per group (*n* = 10) were analyzed using a universal testing machine (K2000 MP, Kratos, Cotia, SP, Brazil) equipped with a 5,000 N load cell. Before testing, the dimensions of each analyzed specimen were measured with a digital caliper.

The specimens were positioned on a three-point bending fixture with a 15.0 mm span between the support cylinders. The load was applied at the center of the specimen at a crosshead speed of 0.5 mm/min until fracture. Three-point flexural strength (*σ*, MPa) was calculated using the following equation:

$$\sigma =3Pl/2wb^{2}$$

where *P* is the fracture load (*N*), l is the support span (mm), *w* is the specimen width (mm), and *b* is the specimen thickness (mm).

### 2.7 Chemical solubility

Chemical solubility was assessed in the second phase by comparing pure HA and the mechanically selected formulation, HA + 1 wt% 3Y-TZP. For each group, 11 disk-shaped specimens were used together to provide the minimum total exposed surface area required by ISO 6872:2024. The 11 disks constituted one gravimetrically measured set per group; they were not treated as 11 independent solubility observations. Before testing, the disks were finished and polished with silicon carbide papers of decreasing abrasiveness (#600, #800, #1,200, #1,500, #2,000, and #2,500), followed by polishing with felt disks and aluminum oxide paste. This procedure was used for surface finishing only; the specimens were not deliberately ground to a target post-sintering thickness. Final dimensions used for surface-area calculations were measured after finishing and immediately before chemical-solubility testing. The measured pre-test dimensions and aggregate chemical-solubility results are reported only in Table 1.

**Table 1:** Pre-test specimen geometry and aggregate chemical-solubility results.

Group	Pre-test diameter (mm)	Pre-test thickness (mm)	Summed surface area, 11 disks (cm^2^)	Aggregate mass loss (mg)	Chemical solubility (µg/cm^2^)
Pure HA	13.00 ± 0.14	1.27 ± 0.04	34.935	73.8	2,112.5
HA + 1 wt% 3Y-TZP	12.09 ± 0.02	1.37 ± 0.03	31.016	63.8	2,057.0

Diameter and thickness are presented as mean ± standard deviation for the 11 disks in each group. Summed surface area, aggregate mass loss, and chemical solubility correspond to one jointly weighed 11-disk gravimetric set per group. Both sets exceeded the ISO 6872:2024 minimum total surface area of 30 cm^2^. No inferential comparison of chemical solubility was performed. Vickers-hardness results are reported only in Figure 5.

The pre-test surface area of each disk was calculated from its individually measured radius and thickness using the formula for the total surface area of a cylinder. The areas of the 11 disks were then summed to obtain the total exposed area of each gravimetrically measured set. The summed areas were 34.935 cm^2^ for pure HA and 31.016 cm^2^ for HA + 1 wt% 3Y-TZP; both therefore exceeded the ISO minimum total surface area of 30 cm^2^, although the HA + 1 wt% 3Y-TZP set was only marginally above this requirement:

$$A=2\pi r^{2}+2\pi rh$$

where *A* is the surface area of one disk, *r* is its radius, *h* is its thickness, and *π* is the mathematical constant. Areas calculated in mm^2^ were converted to cm^2^ by division by 100. Chemical solubility was performed according to ISO 6872:2024 recommendations for dental ceramic materials (20), using the same acetic acid concentration, exposure temperature, and immersion time previously applied to bovine HA-based dental ceramics (8). The 11 disks from each group were ultrasonically cleaned in deionized water for 15 min, dried at 150 ± 5 °C for 4 h in a thermostatically controlled oven, and weighed together using an analytical balance with 0.1 mg readability.

Each set of 11 disks was immersed in 100 mL of 4% acetic acid solution in a sealed 250 mL glass bottle. The bottles were kept in a preheated dry heat oven at 80 ± 2 °C for 16 h. After immersion, the disks were ultrasonically cleaned again in deionized water for 15 min, dried at 150 ± 5 °C for 4 h, and reweighed together. Chemical solubility for each set was calculated as the total mass loss divided by the summed pre-test surface area of its 11 disks:

$$\mathrm{Chemical}\;\mathrm{solubility}(\mu g/cm^{2})=\mathrm{weight}\;\mathrm{loss}(\mu g)/\mathrm{surface}\;\mathrm{area}(cm^{2})$$

### 2.8 Vickers hardness before and after chemical solubility testing

Vickers hardness was measured in pure HA and HA + 1 wt% 3Y-TZP specimens before and after chemical-solubility testing to evaluate local surface indentation response. Six of the eleven disk-shaped specimens used for chemical-solubility testing in each group were evaluated using a Wilson Tukon 1,202 microhardness tester (Buehler, Illinois, USA). A 300 gf load was applied for 15 s, and five indentations were made on each specimen. Their mean provided one experimental-unit value per specimen. Results were reported as Vickers hardness number at 0.3 kgf (HV0.3), mean ± standard deviation; no conversion to GPa was performed. The procedure followed the principles of ISO 14705:2016 (21) and the before/after assessment previously used for bovine HA ceramics subjected to ISO chemical-solubility testing (8). Indentation cracks were not measured, and the test was not used to estimate fracture toughness.

### 2.9 Scanning electron microscopy

SEM was used as a qualitative, complementary method. Retained material from the same nominal W-ZrO_2_ synthesis batch used for specimen fabrication and the as-received commercial 3Y-TZP powder were documented before incorporation into HA (Figure 1). The powder images were acquired between May and October 2023. Representative surface fields of pure HA and HA + 1 wt% 3Y-TZP were also documented before and after chemical-solubility testing (Figures 2, 3). SEM was not used for quantitative dimensional, porosity, roughness, density, phase, or degradation analysis.

**Figure 1:**
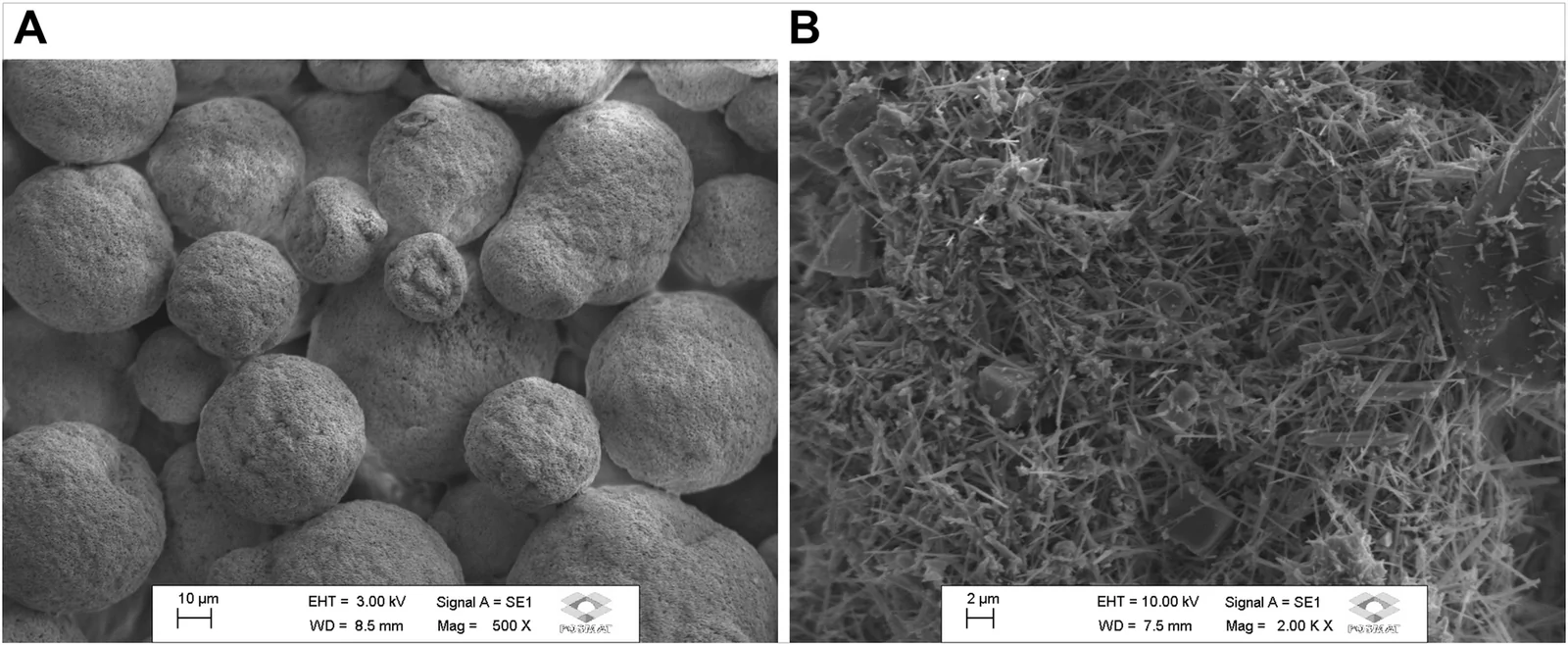
Representative scanning electron microscopy (SEM) images of the as-received commercial 3Y-TZP granulated powder **(A)** and the nominal W-ZrO_2_ synthesis product from the same synthesis batch used for specimen fabrication **(B)**. Panel **A** was acquired at 3.00 kV, WD 8.5 mm, and 500×; panel B at 10.00 kV, WD 7.5 mm, and 2,000×. The different magnifications preclude direct dimensional comparison. Images are presented for qualitative morphological documentation only. Scale bars and acquisition metadata were generated during SEM acquisition and were not modified. SEM does not confirm the phase identity of the nominal W-ZrO_2_ product.

**Figure 2:**
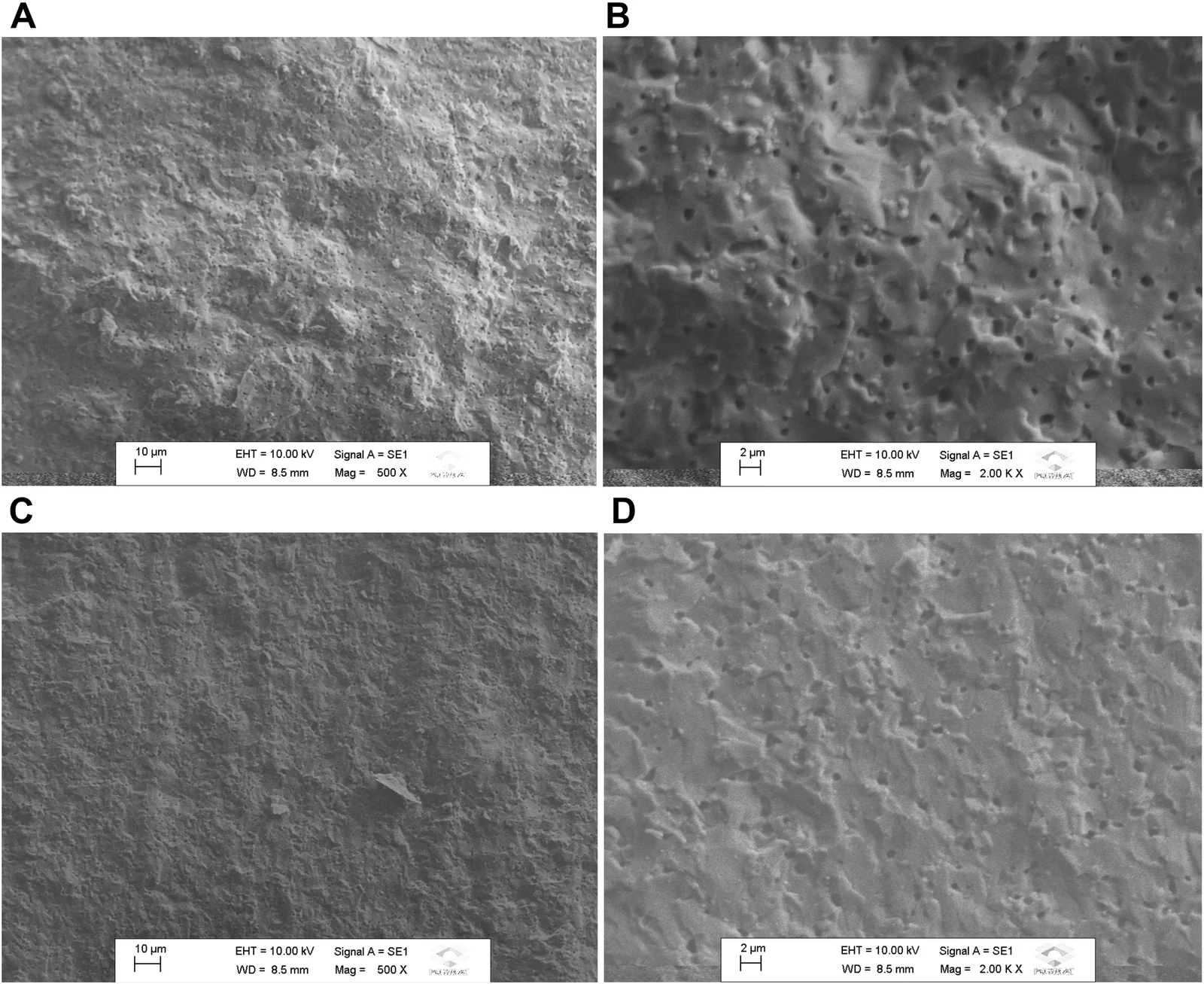
Scanning electron microscopy (SEM) images of pure HA **(A,B)** and HA + 1 wt% 3Y-TZP **(C,D)** before chemical-solubility testing. Images were acquired at 10.0 kV and a working distance of 8.5 mm. Scale bars and acquisition metadata were generated during SEM acquisition and were not modified. Images are interpreted qualitatively; no cross-panel dimensional or inferential image analysis was performed.

**Figure 3:**
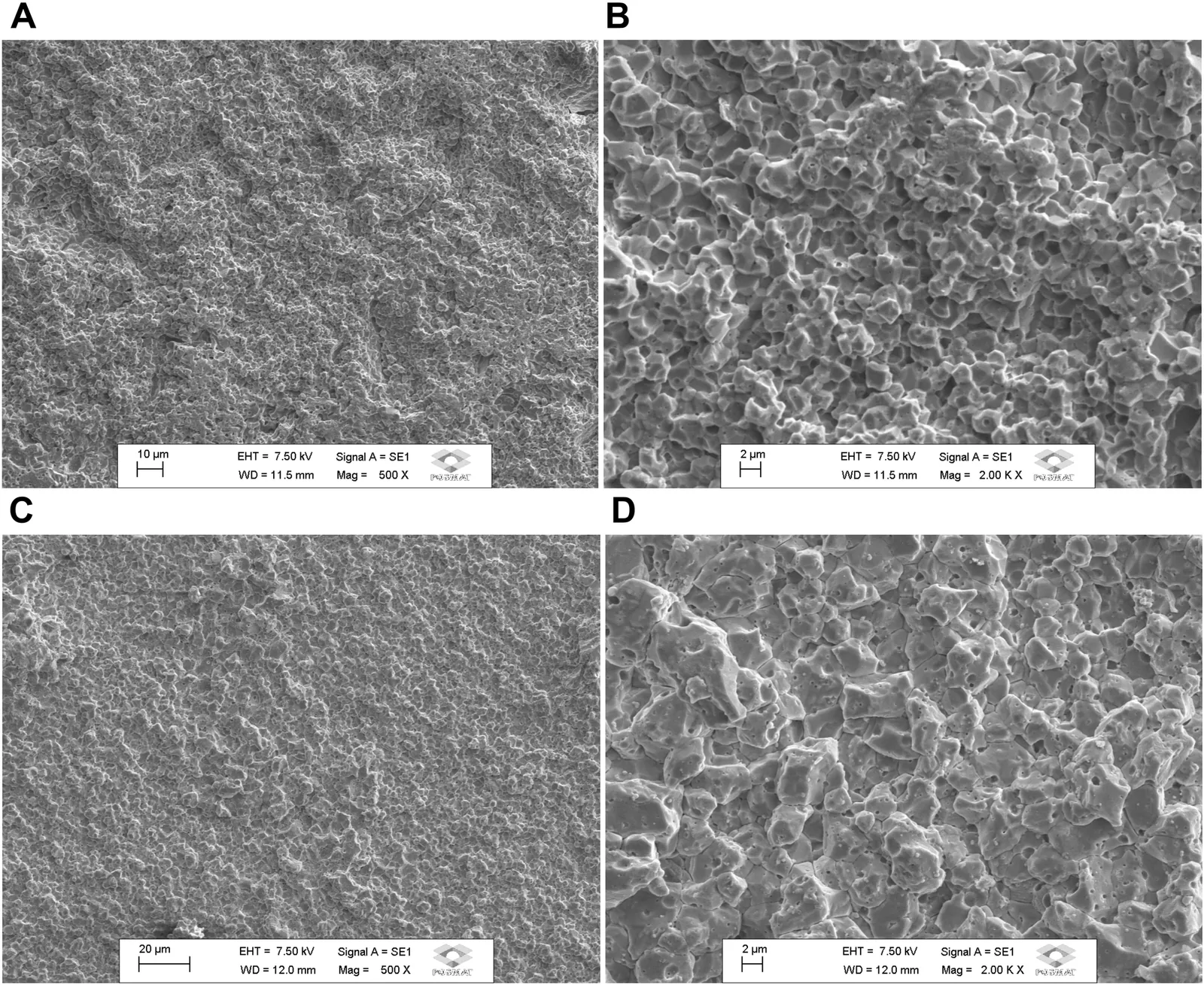
Scanning electron microscopy (SEM) images of pure HA **(A,B)** and HA + 1 wt% 3Y-TZP **(C,D)** after chemical-solubility testing. Images were acquired at 7.5 kV and working distances of 11.5–12.0 mm. Scale bars and acquisition metadata were generated during SEM acquisition and were not modified. Images are interpreted qualitatively; no cross-panel dimensional or inferential image analysis was performed.

The powder additions and representative disk-shaped specimens were mounted on metallic stubs, sputter-coated with gold, and examined using an EVO LS15 scanning electron microscope (Carl Zeiss, Jena, Germany) at the UNESP Bauru multiuser microscopy facility. The as-received commercial 3Y-TZP powder shown in Figure 1A was acquired at 3.00 kV, a working distance of 8.5 mm, and 500× nominal magnification; the nominal W-ZrO_2_ synthesis product shown in Figure 1B was acquired at 10.00 kV, a working distance of 7.5 mm, and 2,000× nominal magnification. Because the powder panels were acquired at different magnifications, they were not used for direct dimensional comparison. The surface fields acquired before chemical-solubility testing (Figure 2) were obtained at 10.0 kV and a working distance of 8.5 mm. The fields acquired after testing (Figure 3) were obtained at 7.5 kV and working distances of 11.5–12.0 mm. Scale bars and acquisition metadata in Figures 1–3 were generated during SEM acquisition and were not modified. The micrographs were selected as representative qualitative fields; no measurements or inferential image analysis were performed.

### 2.10 Statistical analysis

Statistical analyses were performed using jamovi software, version 2.7.37 (The jamovi project, Sydney, Australia). Data distribution was assessed using the Shapiro–Wilk test. Three-point flexural-strength data were analyzed using the Kruskal–Wallis test followed by Dwass–Steel–Critchlow–Fligner (DSCF) pairwise comparisons. Chemical solubility was reported descriptively for each gravimetrically measured set; no inferential comparison was performed because the 11 disks in each group were weighed together and constituted one aggregate observation.

For Vickers hardness, the mean of five indentations was the specimen-level experimental-unit value (*n* = 6 per group). Pre- and post-challenge values were compared separately within each route using two-sided paired *t*-tests, and mean paired changes were reported with 95% confidence intervals. Homogeneity of variance between groups at each time point was assessed using Levene's test; between-group comparisons at each time point were performed using Welch's independent-samples *t*-test. To determine whether the routes differed in their hardness response to the acidic challenge, specimen-level change scores (post-challenge minus pre-challenge) were compared between routes using Welch's independent-samples *t*-test. Bonferroni adjustment was applied across the five inferential hardness comparisons reported (two between-group time-point comparisons, two within-route paired comparisons, and one between-route comparison of change scores). Raw and adjusted *p* values are reported where informative. Flexural strength was reported as median and interquartile range, with mean ± standard deviation additionally provided in Table 2 to permit comparison with the mean-based ISO 6872 strength criteria. Chemical solubility was reported as one descriptive value per gravimetrically measured set, and Vickers hardness as mean and standard deviation. The significance level was *α* = 0.05.

**Table 2:** Three-point flexural strength of the formulation-processing groups.

Group	Median (IQR), MPa	Mean ± SD, MPa
Pure HA	84.0 (77.0, 88.0) ab	83.6 ± 9.2
HA + 1 wt% 3Y-TZP	117.0 (94.8, 122.0) a	110.0 ± 21.8
HA + 5 wt% 3Y-TZP	63.7 (49.8, 80.3) bc	64.0 ± 21.8
HA + 10 wt% 3Y-TZP	24.8 (23.7, 26.7) d	24.4 ± 7.4
HA + 1 wt% nominal W-ZrO_2_	61.5 (60.8, 62.8) c	62.5 ± 5.3
HA + 5 wt% nominal W-ZrO_2_	57.7 (51.8, 60.1) c	56.2 ± 5.1
HA + 10 wt% nominal W-ZrO_2_	27.8 (25.9, 28.7) d	27.6 ± 2.4

Median (IQR) values correspond to the nonparametric analysis; mean ± standard deviation is additionally provided because ISO 6872:2024 flexural-strength class criteria are expressed as minimum mean values. Different lowercase letters indicate DSCF pairwise groupings (*p* < 0.05). No intended clinical class was assigned to these experimental materials. W-ZrO_2_ denotes the nominal, unverified whisker-synthesis product. Because pure HA and zirconia-containing formulations used different sintering schedules, comparisons do not isolate composition from thermal history.

## 3 Results

### 3.1 Three-point flexural strength

Three-point flexural-strength results are reported numerically in Table 2 and graphically as medians and interquartile ranges in Figure 4. Significant differences occurred among formulation-processing groups (Kruskal–Wallis, *p* < 0.001). HA + 1 wt% 3Y-TZP showed the highest observed median [117.0 (94.8, 122.0) MPa]. Its pairwise comparison with pure HA [84.0 (77.0, 88.0) MPa] did not reach statistical significance (DSCF *p* = 0.095). The selected route differed significantly from the remaining zirconia-containing routes (*p* ≤ 0.008). These comparisons describe the complete formulation-processing routes tested and do not isolate composition from sintering schedule.

**Figure 4:**
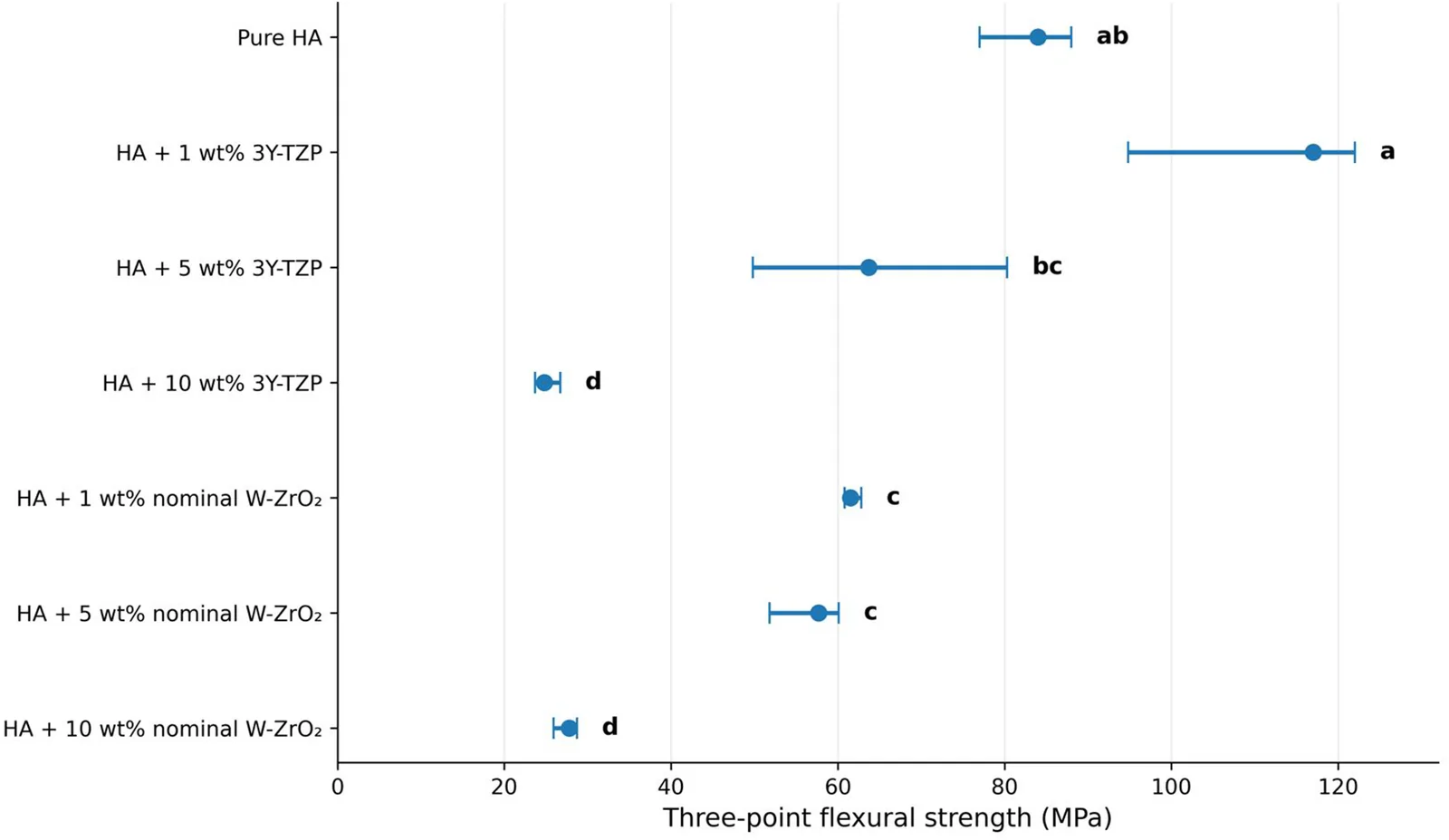
Three-point flexural strength shown as median and interquartile range (IQR). Different lowercase letters indicate the compact-letter grouping derived from Dwass–Steel–Critchlow–Fligner (DSCF) pairwise comparisons following the Kruskal–Wallis test (*p* < 0.05).

HA + 5 wt% 3Y-TZP showed an intermediate median [63.7 (49.8, 80.3) MPa] and was statistically similar to pure HA and to the groups containing 1 and 5 wt% nominal W-ZrO_2_ product. The 1 and 5 wt% nominal W-ZrO_2_ groups showed 61.5 (60.8, 62.8) and 57.7 (51.8, 60.1) MPa, respectively. The lowest values occurred for HA + 10 wt% 3Y-TZP [24.8 (23.7, 26.7) MPa] and HA + 10 wt% nominal W-ZrO_2_ product [27.8 (25.9, 28.7) MPa]; these groups were statistically similar (DSCF *p* = 0.651) and lower than the remaining groups.

### 3.2 Chemical solubility

Chemical-solubility results are reported only in Table 1. The summed pre-test surface areas of the 11-disk sets were 34.935 cm^2^ for pure HA and 31.016 cm^2^ for HA + 1 wt% 3Y-TZP, corresponding to mean per-disk areas of 3.176 and 2.820 cm^2^, respectively. Thus, the mean exposed area per disk was approximately 11.2% smaller for the HA + 1 wt% 3Y-TZP route. Total mass losses were 73.8 and 63.8 mg, respectively, resulting in chemical-solubility values of 2,112.5 μg/cm^2^ for pure HA and 2,057.0 μg/cm^2^ for HA + 1 wt% 3Y-TZP. The zirconia-containing route showed a 55.5 μg/cm^2^ (2.6%) lower descriptive value. Both values exceeded 2,000 μg/cm^2^, the highest chemical-solubility limit specified by ISO 6872:2024 for fully or partially covered substructure ceramics in Classes 2–4; monolithic ceramics in Classes 1–5 are subject to the more stringent <100 μg/cm^2^ limit. No inferential between-group comparison was performed.

### 3.3 Vickers hardness before and after chemical solubility testing

Vickers-hardness results are reported only in Figure 5. Levene's test yielded *p* = 0.059 before and *p* = 0.001 after the acidic challenge. HA + 1 wt% 3Y-TZP showed higher HV0.3 than pure HA both before (435.9 ± 26.4 vs. 217.0 ± 5.6 HV0.3; raw Welch *p* = 2.66 × 10^‒6^, Bonferroni-adjusted *p* < 0.001) and after testing (426.9 ± 38.9 vs. 196.7 ± 10.5 HV0.3; raw Welch *p* = 1.21 × 10^‒5^, adjusted *p* < 0.001).

**Figure 5:**
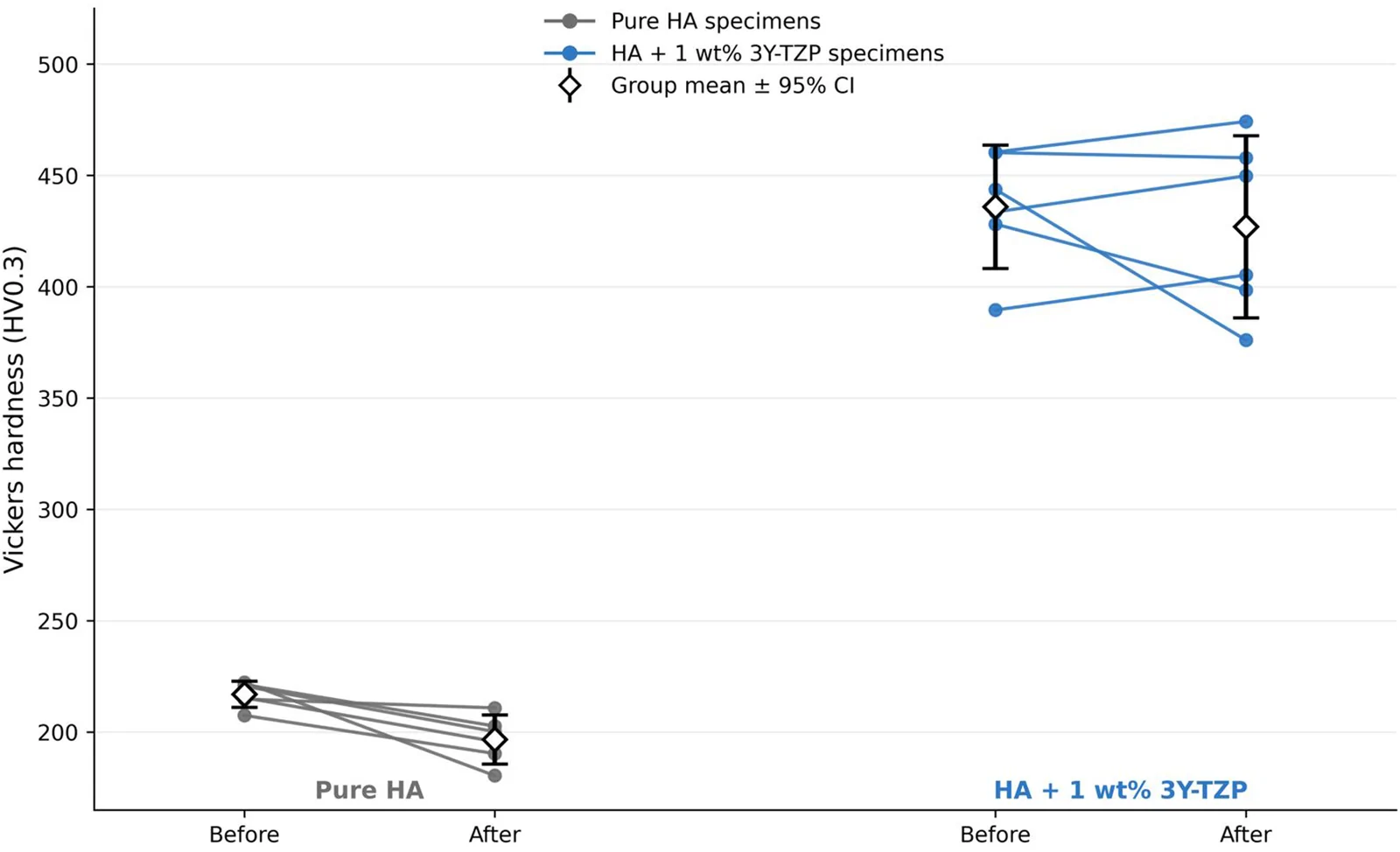
Specimen-level Vickers hardness (HV0.3) before and after chemical-solubility testing. Grey circles/lines indicate pure-HA specimens and blue circles/lines indicate HA + 1 wt% 3Y-TZP specimens. Each point represents the mean of five indentations for one specimen (*n* = 6 per group), and lines connect repeated measurements from the same specimen. Black diamonds indicate group means and error bars indicate 95% confidence intervals. Bonferroni adjustment was applied across the five inferential hardness comparisons reported. HA + 1 wt% 3Y-TZP was harder than pure HA at both time points (adjusted *p* < 0.001). The direct between-route comparison of change scores was not significant (difference, −11.3 HV0.3; 95% CI: −46.7 to 24.1; adjusted *p* = 1.000), and therefore no differential hardness response to the acidic challenge was demonstrated. Chemical-solubility results are not plotted and are reported only in Table 1.

Within pure HA, Vickers hardness decreased by a mean of 20.3 HV0.3 after the acidic challenge (mean paired change, −20.3 HV0.3; 95% CI: −33.1 to −7.5; raw *p* = 0.0097; Bonferroni-adjusted *p* = 0.048). In HA + 1 wt% 3Y-TZP, the mean paired change was −9.0 HV0.3 (95% CI: −44.3 to 26.4; raw *p* = 0.543; adjusted *p* = 1.000). The direct between-route comparison of change scores was not significant (pure HA minus HA + 1 wt% 3Y-TZP, −11.3 HV0.3; 95% CI: −46.7 to 24.1; Welch t = −0.77, df = 6.29; raw *p* = 0.467; adjusted *p* = 1.000). Therefore, the contrast between a significant within-route decrease in pure HA and a non-significant within-route change in HA + 1 wt% 3Y-TZP should not be interpreted as evidence that the routes differ in hardness response to the acidic challenge.

### 3.4 Scanning electron microscopy

Representative SEM images of the as-received commercial 3Y-TZP powder and the nominal W-ZrO_2_ synthesis product are shown in Figures 1A,B, respectively. Figure 1A shows rounded granulated agglomerates at 500×; primary nanoparticles are not resolved at this magnification. Figure 1B shows a morphologically heterogeneous synthesis product at 2,000×, with abundant needle-like/high-aspect-ratio features together with blocky and equiaxed particles. These observations are qualitative. No particle or feature dimensions, aspect-ratio distributions, or phase assignments were derived from the images. The zirconia-based additions were documented before the additional 24-h vibratory homogenization step used for the modified HA powders. Because post-mixing morphology was not evaluated, the starting morphology shown in Figure 1 cannot be assumed to be unchanged after incorporation, in the green bodies, or in the sintered ceramics.

Representative SEM fields before chemical-solubility testing are shown in Figure 2. In the selected fields, pure HA appeared irregular and contained visible pore-like features (Figures 2A,B), whereas HA + 1 wt% 3Y-TZP showed less prominent surface irregularities in the displayed fields (Figures 2C,D). These descriptors are qualitative and do not constitute measurements of pore fraction, roughness, grain size, or group-level morphology.

Representative fields after chemical-solubility testing are shown in Figure 3. The selected pure-HA fields showed an irregular surface with exposed grain-like features (Figures 3A,B), while the selected HA + 1 wt% 3Y-TZP fields appeared visually more continuous (Figures 3C,D). The images are interpreted as representative qualitative fields and do not establish quantitative differences in porosity, density, grain size, or degradation magnitude.

## 4 Discussion

This sequential study ranked seven bovine HA formulation-processing routes by three-point flexural strength and then compared the selected HA + 1 wt% 3Y-TZP route with the pure-HA route by descriptive chemical solubility, repeated Vickers hardness, and qualitative SEM. The flexural-strength screening showed significant overall route differences, although HA + 1 wt% 3Y-TZP did not differ significantly from pure HA in the DSCF pairwise comparison. In Phase 2, the selected route was harder than pure HA at both time points, but the direct comparison of specimen-level hardness changes did not show a differential response to the acidic challenge. Chemical solubility was interpreted descriptively because each group provided one gravimetrically measured 11-disk set. These findings do not establish an isolated zirconia effect because composition and sintering schedule were not independently controlled.

The assessment sequence was selected to answer complementary materials-development questions. Three-point flexural testing provides a standardized, flaw-sensitive screening outcome in the ISO 6872 bar geometry; it does not measure fracture toughness. The ISO acetic-acid protocol provides an accelerated gravimetric assessment of chemical durability, while repeated HV0.3 measurements evaluate whether the local indentation response changes after that challenge. SEM was used only to provide visual surface context. Together, these tools support candidate screening, but they do not by themselves define a clinical indication.

The principal design limitation is the unequal thermal history. The schedules were not selected *ad hoc*: previous optimization supported one-step sintering at 1,300 °C for pure HA and the two-step 1,420 °C route for 3Y-TZP-containing HA (7). Nevertheless, the present experiment does not demonstrate that thermal history affected every measured outcome. Rather, because sintering conditions can influence phase constitution and microstructural features that are relevant to flexural strength, hardness, and dissolution behavior, thermal history is a plausible source of confounding across these route-level comparisons. Its magnitude and direction cannot be determined from the present design. Prior optimization justifies why the routes were selected but does not replace same-schedule bridging controls; causal language attributing current differences specifically to composition or processing is therefore not warranted.

The non-linear strength ranking is empirically clear, and the authors' prior investigation provides directly relevant, system-specific phase evidence. In sintered specimens produced from the same starting powder lot and within the same nominal composition-processing framework, XRD showed characteristic HA reflections in pure HA and HA + 1 wt% 3Y-TZP, whereas HA decomposition to β-TCP and calcium zirconate formation were detected at 5 and 10 wt% 3Y-TZP (7). These findings provide a stronger basis for interpreting the present ranking than evidence from unrelated HA-zirconia systems and are consistent with the composition- and temperature-dependent reactions reported elsewhere (13, 14). However, the specimens evaluated here were newly manufactured and were not subjected to XRD/Rietveld analysis. The prior phase data are therefore treated as contextual evidence and cannot be assigned as measurements of the current specimens or used to establish their exact phase fractions.

Likewise, relative density, open porosity, and pore-size distribution were not measured. Relative density was not an outcome of the present experimental design, and dedicated specimens were therefore not prepared for that measurement. Flexural strength in brittle ceramics is sensitive to the flaw population, but the observed ranking cannot be attributed to densification, residual porosity, packing heterogeneity, or grain size without direct measurements. The lower strength at 5 and 10 wt% 3Y-TZP should therefore be described as an association under the tested route rather than evidence of a specific defect-generating mechanism.

SEM of the starting additions documented qualitative morphological differences between the as-received commercial 3Y-TZP powder and the nominal W-ZrO_2_ synthesis product (Figure 1). The 3Y-TZP image shows granulated agglomerates rather than resolved primary nanoparticles, whereas the nominal W-ZrO_2_ product contains both needle-like features and blocky/equiaxed particles, indicating morphological heterogeneity. SEM alone does not establish chemical or crystalline phase identity. Moreover, the precursor quantities used for the nominal W-ZrO_2_ synthesis correspond to a P:Zr molar ratio of approximately 0.85; therefore, phosphate-bearing zirconium phases or residual salt-derived products cannot be excluded solely from morphology. The additions were imaged before the additional 24-h vibratory homogenization step used to distribute them within HA. Because the morphology was not re-evaluated after this processing step, persistence of the starting morphology in the green or sintered bodies cannot be assumed. The flexural-strength findings for these groups are therefore interpreted only as exploratory synthesis-and-processing-route results, not as evidence of verified whisker reinforcement or whisker-toughening. Powder XRD remains necessary for phase confirmation.

The incremental contribution should be interpreted in relation to the authors' prior work. Using the same bovine-HA starting powder lot, Azevedo-Silva et al. (7) established composition-sintering relationships through XRD, biaxial flexural strength, Vickers hardness, and microstructural assessment. The current specimens were manufactured anew, and the present outcome dataset is independent. The present contribution comprises three-point flexural-strength screening in ISO bar geometry, exploratory comparison of 3Y-TZP routes with routes containing the nominal W-ZrO_2_ synthesis product, and application of the ISO chemical-solubility and repeated-hardness workflow to the route selected on the basis of the highest observed median flexural strength.

Chemical solubility was recalculated from the archived raw measurements using the total mass loss and summed pre-test area of each 11-disk set. Pure HA yielded 2,112.5 μg/cm^2^ and HA + 1 wt% 3Y-TZP yielded 2,057.0 μg/cm^2^, a descriptive difference of 55.5 μg/cm^2^ (2.6%). Both sets satisfied the ISO minimum total surface area of 30 cm^2^, although the HA + 1 wt% 3Y-TZP set was only marginally above it (31.016 cm^2^). Importantly, the post-sintering/pre-test geometry differed between routes: the mean per-disk surface area was 3.176 cm^2^ for pure HA and 2.820 cm^2^ for HA + 1 wt% 3Y-TZP, approximately 11.2% lower in the selected route. This difference is consistent with route-specific shrinkage but cannot be causally assigned to composition or thermal history in the present design. Specimen geometry can influence the outcome and reproducibility of the ISO chemical-solubility test (22). Accordingly, the small 2.6% difference is presented only descriptively and should not be interpreted as evidence that the selected route intrinsically reduced solubility. Because the disks within each group were weighed together, they formed one gravimetric unit rather than independent replicates; inferential comparison was therefore not performed.

The specimen-level hardness reanalysis retained each disk as the experimental unit and used the mean of five indentations per specimen. Pure HA showed a within-route decrease of −20.3 HV0.3 (95% CI: −33.1 to −7.5; adjusted *p* = 0.048), whereas HA + 1 wt% 3Y-TZP showed a change of −9.0 HV0.3 (95% CI: −44.3 to 26.4; adjusted *p* = 1.000). Critically, the direct comparison of these change scores was not significant (difference, −11.3 HV0.3; 95% CI: −46.7 to 24.1; adjusted *p* = 1.000). Thus, the different significance results of the two separate within-route tests do not demonstrate that the selected route was more resistant to the acidic challenge. HA + 1 wt% 3Y-TZP remained harder than pure HA at both measured time points, but this represents a difference in hardness level rather than evidence of a differential hardness response to acid exposure. Flexural strength and hardness do not measure fracture toughness or demonstrate transformation toughening, and calibrated radial-crack lengths were not measured (23).

SEM findings were intentionally descriptive. Figure 1 documents the qualitative morphology of the two zirconia-based starting additions, whereas Figures 2, 3 show representative surface fields before and after the acidic challenge. The powder panels were acquired at different magnifications, and the pre- and post-challenge specimen sessions also used different accelerating voltages and working distances; these parameters are explicitly reported in the Methods and captions. Scale bars and acquisition metadata were generated during acquisition and were not modified. Because no particle-size, grain-size, porosity, roughness, or degradation measurements were performed, the images were not subjected to quantitative cross-panel comparison or inferential analysis.

Within these limits, the non-linear route ranking remains useful for screening. HA + 1 wt% 3Y-TZP had the highest observed flexural-strength median, although it was not significantly different from pure HA in the DSCF pairwise comparison. Phase 2 showed that the selected route was harder than pure HA at both time points; however, direct comparison of the pre-to-post hardness changes did not demonstrate a differential response to the acidic challenge. Chemical solubility differed by only 2.6% descriptively, and this small difference must be interpreted in light of the unequal specimen geometry and the single aggregate gravimetric set per group. The appropriate inference is that this complete route warrants controlled follow-up, not that 1 wt% 3Y-TZP is an established composition-only optimum or that it confers superior acid resistance.

As designed, this study was intended as route-level screening rather than as a full mechanistic or application-validation investigation. Composition and sintering schedule were not independently isolated, the nominal W-ZrO_2_ product was not phase-verified, and Phase 2 was limited to chemical solubility, Vickers hardness, and qualitative SEM assessment of the selected route vs. pure HA. Relative density was not part of the experimental design, and dedicated specimens were not prepared for that measurement. These boundaries should be considered when interpreting the results.

Accordingly, the present data identify a screening candidate for controlled follow-up rather than a clinically validated dental ceramic. Future studies should use harmonized thermal controls and targeted phase, density, microstructural, mechanical, and chemical characterization appropriate to the specific application under investigation.

## 5 Conclusions

Among the formulation-processing routes tested, HA + 1 wt% 3Y-TZP showed the highest observed three-point flexural-strength median; however, its pairwise comparison with pure HA was not statistically significant (DSCF *p* = 0.095). The selected route showed higher HV0.3 than pure HA before and after the acidic challenge, but the direct comparison of pre-to-post hardness changes was not significant (difference, −11.3 HV0.3; 95% CI: −46.7 to 24.1; adjusted *p* = 1.000). Therefore, the present data do not demonstrate a differential hardness response to the acidic challenge. Descriptive chemical solubility was 2,112.5 μg/cm^2^ for pure HA and 2,057.0 μg/cm^2^ for HA + 1 wt% 3Y-TZP; both exceeded the <2,000 μg/cm^2^ limit applicable to covered substructure ceramics in ISO 6872:2024 Classes 2–4, and also the more stringent <100 μg/cm^2^ limit for monolithic ceramics. Because the solubility sets differed in post-sintering geometry and each group contributed one aggregate gravimetric observation, the 2.6% difference should not be interpreted as evidence of improved chemical durability.

The HA + 1 wt% 3Y-TZP formulation-processing route should be regarded as an experimental screening candidate. The nominal W-ZrO_2_ routes remain exploratory because phase identity and post-homogenization morphology were not verified. Because no common-schedule bridging control was included, differences cannot be attributed to 3Y-TZP independently of thermal history. Further application-specific development requires controlled phase, density, microstructural, mechanical, chemical, and biological characterization.

## Data Availability

The raw data supporting the conclusions of this article will be made available by the authors, without undue reservation.
